# Micromolar Valproic Acid Doses Preserve Survival and Induce Molecular Alterations in Neurodevelopmental Genes in Two Strains of Zebrafish Larvae

**DOI:** 10.3390/biom10101364

**Published:** 2020-09-24

**Authors:** Andrea Messina, Alessandra Boiti, Valeria Anna Sovrano, Paola Sgadò

**Affiliations:** 1 Center for Mind/Brain Sciences, University of Trento, 38068 Rovereto, Italy; alessandraboiti@gmail.com (A.B.); valeriaanna.sovrano@unitn.it (V.A.S.); 2 Department of Psychology and Cognitive Science, University of Trento, 38068 Rovereto, Italy

**Keywords:** VPA, AB inbred zebrafish strain, TU inbred zebrafish strain, serotonin, dopamine, *ascl1a/b*

## Abstract

Autism spectrum disorders (ASDs) comprise a genetically heterogeneous group of conditions characterized by a multifaceted range of impairments and multifactorial etiology. Epidemiological studies have identified valproic acid (VPA), an anticonvulsant used to treat epilepsy, as an environmental factor for ASDs. Based on these observations, studies using embryonic exposure to VPA have been conducted in many vertebrate species to model ASD. The zebrafish is emerging as a popular model in biomedical research to study the molecular pathways involved in nervous system disorders. VPA exposure in zebrafish larvae has been shown to produce a plethora of effects on social, motor and anxiety behavior, and several genetic pathways altered by VPA have been described. However, the doses and regimen of administration reported in the literature are very heterogenous, creating contradictory results and posing serious limits to the interpretation of VPA action on neurodevelopment. To shed light on the toxic effect of VPA, we tested micromolar concentrations of VPA, using exposure for 24 and 48 h in two different zebrafish strains. Our results show that micromolar doses of VPA mildly affect embryo survival but are sufficient to induce molecular alterations in neurodevelopmental genes previously shown to be influenced by VPA, with substantial differences between strains.

## 1. Introduction

Autism spectrum disorders (ASDs) comprise a multifaceted, genetically heterogeneous group of neurodevelopmental conditions characterized by a range of behavioral impairments, including deficits in social interaction, repetitive behavior and increased sensitivity to sensory stimuli. ASDs have a complex and multifactorial etiology, involving genetic variations in several hundred genes as well as environmental factors. Prospective and retrospective clinical studies have identified valproic acid (VPA) as an environmental factor for ASDs. VPA is an anticonvulsant used to treat epilepsy, also used as a mood stabilizer to treat bipolar disorder, whose embryonic exposure has been associated with several instances of an increased risk for ASDs [1,2,3]. Based on these observations, studies using embryonic exposure to VPA have also been conducted in many vertebrate species to model the core signs of ASDs [4,5,6,7,8].

Several studies have analyzed social behavioral deficits in rodents prenatally exposed to VPA, describing lifelong impairments resembling the core signs of ASDs [9]. In rats, a single administration of VPA in utero induces gender-specific social behavior abnormalities, modifies sensitivity to sensory stimuli, and increases repetitive behavior and anxiety [4,5,6,10]. Mice exposed to prenatal VPA also show cellular and molecular phenotypes in the medial prefrontal cortex [11], the somatosensory cortex, the amygdala [12], the brain stem and the cerebellum [13]. In domestic chicks, VPA has been shown to produce alterations in early social-orienting responses and social interaction [7,8,14].

The zebrafish is emerging as a popular model in biomedical research to study the effect of neurotoxicants involved in nervous system disorders. VPA exposure in zebrafish larvae has been shown to produce a plethora of effects on social behavior, locomotor activity and anxiety. Zimmerman et al. [15] have reported changes in locomotor activity, increased anxiety and social interaction deficits in zebrafish larvae exposed to 48 µM VPA for 48 h. Liu et al. [16] exposed 24 hpf zebrafish larvae to chronic (20 µM for 6 days) or acute (100 µM for 7 h) VPA treatment, reporting altered locomotor activity and social preference deficits. Molecular studies have also been conducted in VPA exposed zebrafish embryos to assess the neurobiological effect of VPA exposure. A recent study from Dwivedi et al. [17] has reported altered levels of ASD-associated gene and protein expression in the brains of zebrafish larvae treated with 75 µM VPA for 5 days, accompanied by increased anxiety, social deficits and high circling behavior.

Histone deacetylases (HDACs) inhibition mediated by VPA has also been shown to directly inhibit *ascl1b* expression, causing the selective failure of serotonergic identity [18], a reduction of histaminergic neurons, changes in the expression of key genes of other monoaminergic systems, including dopamine, and decreased adult brain levels of noradrenaline and dopamine metabolites [19].

Overall, the emerging picture on VPA exposure in zebrafish is very heterogeneous, with different doses, time and duration of exposure being reported. This poses serious limits to the interpretation of VPA action on neurodevelopment and to the use of the VPA zebrafish model to study ASD-relevant features.

To shed light on the neurotoxic effect of VPA on zebrafish larvae, we tested different micromolar concentrations of VPA, using exposure for 24 and 48 h in two different zebrafish inbred strains. Our data shows that, at 1 µM concentration, VPA mildly affects embryo survival and induces molecular alterations in neurodevelopmental genes, previously reported only using higher VPA doses, with substantial differences between strains.

## 2. Materials and Methods

### 2.1. Ethical Regulations

All husbandry and experimental procedures complied with the European Directive 2010/63/EU on the protection of animals used for scientific purposes and were approved by the Scientific Committee on Animal Health and Animal Welfare (Organismo Preposto al Benessere Animale, OPBA) of the University of Trento and by the Italian Ministry of Health (Protocol n. 135/2020-PR).

### 2.2. Animals

Adult AB and TU zebrafish were placed in tanks overnight. Males and females were separated by a transparent barrier that was removed the following morning for breeding. After collection, embryos were transferred in E3 medium (5.00 mM NaCl, 0.44 mM CaCl2, 0.33 mM MgSO4 and 0.17 mM KCl) and were staged according to Kimmel et al. [20]. At 5 hpf, an average of 100 embryos for each treatment group were transferred in 10 cm Petri dishes containing E3 medium (control) and E3 medium with 1, 2.5 and 5 μM VPA. Embryos were incubated in VPA (Sigma-Aldrich, P4543; Merck Life Science Srl, Milan, Italy) solutions for 24 or 48 h, and then the medium was replaced by E3 medium. Embryos were grown at 28.5 °C.

### 2.3. Survival Analysis

Embryos exposed to VPA or the control solution were observed daily until 5 dpf. The number of embryos were counted under a stereomicroscope at 48, 96 and 120 h for the group treated with different VPA concentrations and at 24, 48, 96 and 120 hpf for the 1 µM treatment group.

### 2.4. RNA Extraction

Total RNA extraction was performed using the RNeasy Mini Kit (QIAGEN; Milan, Italy) according to the manufacturer’s instructions. Briefly, a pool of 15 embryos were collected from each treatment at the different time points (2 and 5 dpf) and homogenized in lysis buffer. Lysates were loaded onto RNeasy spin columns, treated with DNase (RNase-Free DNase Set, QIAGEN; Milan, Italy) and eluted in RNase/DNase-free water. Collected total RNAs were quantified using NanoDrop™ (Thermo Fisher Scientific; Monza, Italy), and reverse transcriptions were performed using the SuperScript™ VILO™ cDNA Synthesis Kit (Invitrogen, Thermo Fisher Scientific; Monza, Italy) according to manufacturer’s instructions.

### 2.5. Reverse Transcription—Quantitative Polymerase Chain Reaction (RT-qPCR)

The RT-qPCR experiments were performed using specific, commercially synthesized primer pairs (Merck Life Science Srl, Milan, Italy). Primers used for RT-qPCR are listed in Table S1. The triplicate reactions/samples were performed using the PowerUp™ SYBR™ Green Master Mix (2×) and a CFX96™ Real-Time System (Bio-Rad, Milan, Italy). The DCT method was used for expression quantification [21]. Data were normalized on the expression of the 18S reference gene.

### 2.6. Statistical Analysis

Statistical evaluation of the effect of treatment on zebrafish larvae survival was assessed using the Mantel-Cox log-rank test using Prism 8.4.3 (GraphPad Software, San Diego, CA, USA). Statistical evaluation of the log2 gene expression levels (dCt) was assessed using mixed-effect models with random slopes and intercepts to account for interactions between the fixed factors (treatment, age and transcript) and the random factor (experiment), using the nlme package in R (https://cran.r-project.org/web/packages/nlme/index.html). For Tukey mean comparison tests, we used the emmeans package in R (https://cran.r-project.org/web/packages/emmeans/index.html).

## 3. Results

**Figure 1:**
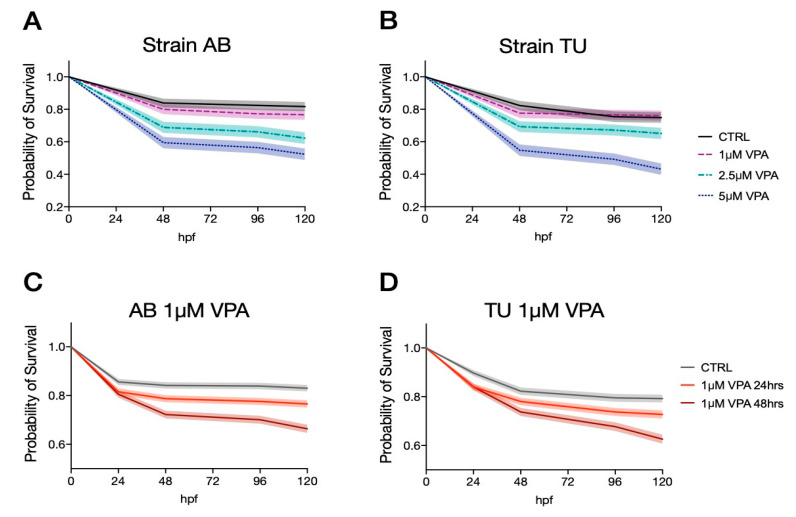
Effect of valproic acid (VPA) exposure on zebrafish embryo survival. (**A**,**B**) Kaplan–Meier survival comparison for groups of treatment showing a significant effect (log-rank (Mantel-Cox) test) of VPA treatment at 1, 2.5 and 5 µM concentration in AB (**A**) and TU (**B**) strains. (**C**,**D**) Kaplan-Meier survival comparison showing a significant effect (log-rank (Mantel-Cox) test) of 1 µM VPA exposure for 24 and 48 h in AB (**C**) and TU (**D**). Probability of survival with shaded color indicating SE.

In order to identify the doses and administration regimens that have the lowest neurotoxic effects, we treated zebrafish larvae from two inbred strains (AB and TU) with different doses of VPA (1, 2.5 and 5 µM VPA) in five independent experiments (n = 735 and n = 756 for AB and TU, respectively). We administered 1, 2.5 and 5 µM VPA to zebrafish larvae for 24 h, starting from 5 hpf. The survival rates were analyzed at different time points (48, 96 and 120 hpf) using the log-rank (Mantel-Cox) test. AB zebrafish larvae treated with the vehicle showed a survival rate of 0.839 [95% C.I. 0.046, 0.062] at 48 hpf and at 96 hpf, and 0.817 [95% C.I. 0.049, 0.065] at 120 hpf. The survival rate for the three VPA concentrations (1 µM VPA: 0.800 [95% C.I. 0.051, 0.066] at 48 hpf, 0.772 [95% C.I. 0.055, 0.068] at 96 hpf and 0.767 [95% C.I. 0.055, 0.069] at 120 hpf; 2.5 µM VPA: 0.689 [95% C.I. 0.062, 0.073] at 48 hpf, 0.661 [95% C.I. 0.064, 0.074] at 96 hpf and 0.622 [95% C.I. 0.066, 0.075]; 5 µM VPA: 0.595 [95% C.I. 0.065, 0.072] at 48 hpf, 0.564 [95% C.I. 0.066, 0.073] at 96 hpf and 0.523 [95% C.I. 0.067, 0.072]) were significantly different from the vehicle-treated controls, as indicated by the log-rank (Mantel-Cox) test (Figure 1A, *χ*^2^_(3,735)_ = 59.63, *p* < 0.0001).

The vehicle treated TU larvae survival rates were 0.823 [95% C.I. 0.049, 0.065] at 48 hpf, 0.754 [95% C.I. 0.057, 0.071] at 96 hpf and 0.749 [95% C.I. 0.058, 0.071] at 120 hpf. Valproic acid treatment induced a significant reduction of the survival rates in all three concentrations (Figure 1B, log-rank (Mantel-Cox) test *χ*^2^_(3,756)_ = 46.60, *p* < 0.0001). The survival rates at the different concentration and time points were as follows: 1 µM VPA 0.776 [95% C.I. 0053, 0.066] at 48 and 96 hpf and 0.760 [95% C.I. 0.054, 0.067] at 120 hpf; 2.5 µM VPA 0.693 [95% C.I. 0.060, 0.071] at 48 hpf, 0.672 [95% C.I. 0.061, 0.071] at 96 hpf and 0.651 [95% C.I. 0.063, 0.072] at 120 hpf; 5 µM VPA 0.584 [95% C.I. 0.066, 0.072] at 48 hpf, 0.492 [95% C.I. 0.068, 0.072] at 96 hpf and 0.431 [95% C.I. 0.068, 0.070] at 120 hpf.

Overall, valproic acid seems to produce a similar toxic effect on the two strains, as indicated by the significant effect of VPA treatments in both strains. The two strains seem to have a similar survival rate in the vehicle-treated samples at 48 hpf but tend to diverge at later time points, especially when treated with the highest VPA concentration (log-rank (Mantel-Cox) test between AB and TU 5 µM VPA, *χ*^2^_(1,392)_ = 3.0770 uncorrected *p* = 0.0794, data not shown).

Given the similar neurotoxic effect of the different VPA concentrations, we evaluated the lowest tested concentrations using two distinct regimens: treatment for 24 or 48 h. The survival rates for zebrafish larvae treated with the two regimens were similar in both strains, as indicated in Figure 1C,D, representative of AB and TU strain, respectively. The survival rates for AB were for vehicle-treated larvae 0.856 [95% C.I. 0023, 0.027] at 24 hpf, 0.842 [95% C.I. 0.024, 0.028] at 48 hpf, 0.839 [95% C.I. 0.024, 0.028] at 96 hpf and 0.830 [95% C.I. 0.025, 0.028] at 120 hpf; 1 µM VPA for 24 h 0.815 [95% C.I. 0.025, 0.029] at 24 hpf, 0.787 [95% C.I. 0.027, 0.030] at 48 hpf, 0.776 [95% C.I. 0.028, 0.031] at 96 hpf and 0.766 [95% C.I. 0.028, 0.031] at 120 hpf; 1 µM VPA for 48 h 0.805 [95% C.I. 0.026, 0.029] at 24 hpf, 0.722 [95% C.I. 0.030, 0.033] at 48 hpf, 0.701 [95% C.I. 0.031, 0.033] at 96 hpf and 0.664 [95% C.I. 0.032, 0.034] at 120 hpf. Log-rank (Mantel-Cox) test indicated a significant effect of VPA treatment for both regimens compared to the vehicle (Figure 1C, log-rank (Mantel-Cox) test *χ*^2^_(2, 2340)_ = 55.93, *p* < 0.0001).

TU strain larvae showed comparable survival rates: vehicle-treated larvae 0.897 [95% C.I. 0021, 0.027] at 24 hpf, 0.823 [95% C.I. 0028, 0.032] at 48 hpf, 0.795 [95% C.I. 0030, 0.034] at 96 hpf and 0.792 [95% C.I. 0030, 0.034] at 120 hpf; 1 µM VPA for 24 h 0.840 [95% C.I. 0.025, 0.029] at 24 hpf, 0.780 [95% C.I. 0.029, 0.033] at 48 hpf, 0.737 [95% C.I. 0.031, 0.034] at 96 hpf and 0.727 [95% C.I. 0.031, 0.035] at 120 hpf; 1 µM VPA for 48 h 0.840 [95% C.I. 0.025, 0.029] at 24 hpf, 0.737 [95% C.I. 0.031, 0.034] at 48 hpf, 0.677 [95% C.I. 0.033, 0.036] at 96 hpf and 0.626 [95% C.I. 0.035, 0.037] at 120 hpf. As for AB strain larvae, a significant difference was observed in the survival rates among treatment regimens (Figure 1D, log-rank (Mantel-Cox) test *χ*^2^_(2, 2020)_ = 42.10, *p* < 0.0001).

Direct comparison between survival rates of embryos exposed to VPA for 24 and 48 h indicated a significant effect of treatment regimens in both strains (Figure 1C,D, log-rank (Mantel-Cox) test: *χ*^2^_(1, 1570)_ = 18.19, uncorrected *p* < 0.0001 for AB and *χ*^2^_(1, 1400)_ = 14.02, uncorrected *p* < 0.0002 for TU).

To assess the neurodevelopmental effects of VPA treatments, we compared the expression levels of genes previously shown to be affected by VPA treatment at higher dosage and different treatment regimens. To assess the effect of treatment, age and transcript on gene expression levels, we used a linear mixed model (LMM), considering treatment, age and transcript as fixed factors and the experimental unit (experiment) as random factor. We compared a model with random-intercepts only to one with random slopes and intercepts, using the likelihood ratio, and found that the random slopes and intercepts approach fitted the data significantly better.

Data from the two zebrafish inbred strains AB and TU were analyzed separately. We found that gene expression was significantly affected by treatment, age and transcript in both strains (LMM for AB: treatment *χ*^2^(2) = 30.78, *p* < 0.0001; age *χ*^2^(1) = 9.04, *p* = 0.0026; transcript *χ*^2^(9) = 1216.56, *p* < 0.0001. LMM for TU: treatment *χ^2^*(2) = 7.05, *p* = 0.0295; age *χ*^2^(1) = 38.11, *p* < 0.0001; transcript *χ*^2^(9) = 492.79, *p* < 0.0001). Significant interactions between the fixed factors were also assessed in both strains, in particular a triple interaction between treatment, age and transcript was observed for both AB and TU zebrafish larvae (LMM for AB: treatment*age *χ*^2^(2) = 1.84, *p* = 0.3978; treatment*transcript *χ*^2^(18) = 17.52, *p* = 0.4875; age*transcript: *χ*^2^(9) = 282.88, *p* < 0.0001; treatment*age*transcript: *χ*^2^(18) = 37.91, *p* = 0.0040. LMM for TU: treatment*age *χ*^2^(2) = 30.88, *p* < 0.0001; treatment*transcript *χ*^2^(18) = 26.54, *p* = 0.0880; age*transcript: *χ*^2^(9) = 580.06, *p* < 0.0001; treatment*age*transcript: *χ*^2^(18) = 56.13, *p* < 0.0001).

**Figure 2:**
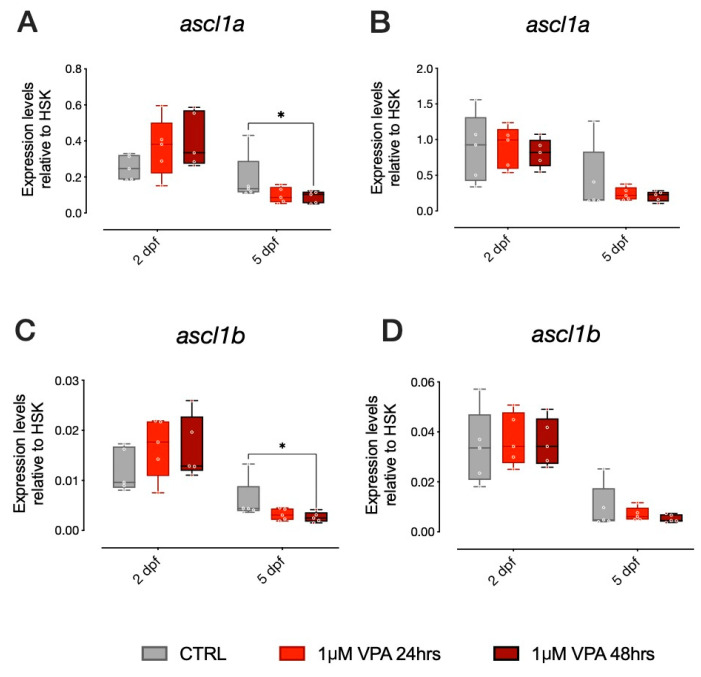
mRNA expression of *ascl1a* and *ascl1b* in 2 dpf and 5 dpf AB and TU zebrafish after 24 or 48 h of 1 µM VPA. Changes in the expression of *ascl1a* (**A**,**B**) and *ascl1b* (**C**,**D**) in AB (**A**,**C**) and TU (**B**,**D**) zebrafish embryos. Box plot (median, min to max) of mean dCt values for each group. **** *p* < 0.0001, *** *p* < 0.001, ** *p* < 0.01, * *p* < 0.05.

VPA has been shown to impair development of the 5HT system acting on 5HT neuronal progenitor differentiation [18]. Therefore, we examined the effect of minimal doses of VPA (1 µM) on genes involved in 5HT neuron differentiation in the two different strains. *ascl1a* and *ascl1b* are bHLH transcription factors, homologs to the mammalian *Ascl1* (also known as *Mash1*) involved in neural differentiation, that have been shown to be direct targets of VPA and to affect 5HT progenitor specification [18]. In the AB strain larvae, we found that gene expression levels for *ascl1a* were different in vehicle-treated control larvae at 5 dpf compared to larvae treated with 1 µM VPA for 48 h, as shown in Figure 2A (Tukey post-hoc pairwise comparison at 5 dpf: CTRL-1 µM VPA for 48 h t_(236)_ = 2.528, *p* = 0.0324). For *ascl1b*, we found a similar effect of treatment in the AB strain larvae. The *ascl1b* gene expression levels were different from vehicle-treated control larvae at 5 dpf in larvae treated with 1 µM VPA for 48 h (Figure 2C; Tukey post-hoc pairwise comparison at 5 dpf: CTRL-1 µM VPA for 48 h t_(226)_ = 2.839, *p* = 0.0136). Thus, exposure to 1 µM VPA for 48 h seems to be sufficient to affect the expression of proneural genes involved in differentiation and specification of progenitor neurons in the AB strain. In the TU strain, however, VPA did not produce the same effect on *ascl1a* and *ascl1b*. We did not observe any significant difference in the expression levels of *ascl1a* or *ascl1b* in the TU strain (Figure 2B,D, respectively).

Notice that our data also indicates that *ascl1a* and *ascl1b* expression is significantly affected by age in both AB and TU strains, as it decreases between 2 and 5 dpf, independent of VPA treatment. Overall, we observed a different effect of VPA treatment at 1 µM concentration in the two zebrafish strains, as *ascl1a* and *ascl1b* expression seemed to be affected by VPA treatment only in larvae from the AB strain.

**Figure 3:**
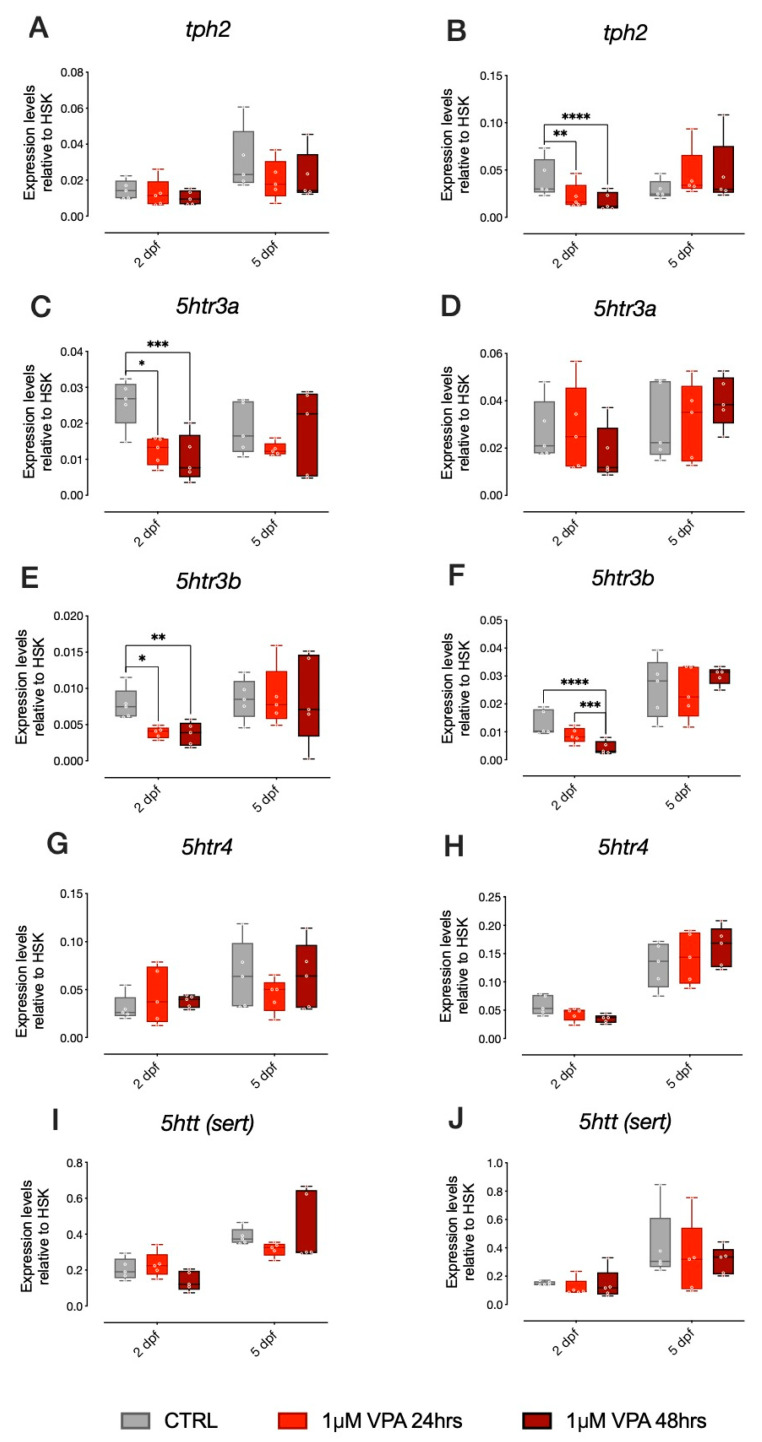
mRNA expression of key genes of serotoninergic system in 2 dpf and 5 dpf AB and TU zebrafish after 24 or 48 h of 1 µM VPA. Changes in the expression of *tph2* (**A**,**B**), *5htr3a* (**C**,**D**), *5htr3b* (**E**,**F**), *5htr4* (**G**,**H**) and *5htt (sert)* (**I**,**J**) in AB (**A**,**C**,**E**,**G**,**I**) and TU (**B**,**D**,**F**,**H**,**J**) zebrafish embryos. Box plot (median, min to max) of mean dCt values for each group. **** *p* < 0.0001, *** *p* < 0.001, ** *p* < 0.01, * *p* < 0.05.

VPA-mediated changes in *ascl1a* and *ascl1b* have been previously shown to affect the brain serotonin system through direct inhibition of serotonin progenitor differentiation. We thus analyzed the expression of *tph2*, the rate-limiting enzyme for brain serotonin synthesis, as well as other genes involved in serotonergic neurotransmission, such as the serotonin transporter (*sert*) and three of the most important serotonin receptors (*5htr3a, 5htr3b* and *5ht4*). For *tph2*, we did not observe any significant effect of treatment in AB strain larvae (Figure 3A). Despite the absence of significant changes in *ascl1a* and *ascl1b* expression levels, in the TU strain larvae, we observed that *tph2* expression levels were significantly affected by our VPA treatment regimen, but only at 2 dpf (Figure 3B; Tukey post-hoc pairwise comparison at 2 dpf: CTRL-1 µM VPA 24 h t_(236)_ = 2.978, *p* = 0.0090; CTRL-1 µM VPA 48 h F_(2, 8)_ = 4.230, *p* = 0.0001).

Concerning the expression of 5HT receptors, in AB strain larvae, *5htr3a* expression levels were significantly affected by treatment at 2 dpf (Figure 3C; Tukey post-hoc pairwise comparison at 2 dpf: CTRL-1 µM VPA 24 h t_(236)_ = 2.898, *p* = 0.0114; CTRL-1 µM VPA 48 h t_(236)_ = 4.087, *p* = 0.0002). In the TU strain larvae, *5htr3a* expression levels were not significantly affected by treatment (Figure 3D). For *5htr3b* we observed a significant effect of treatment in AB strain larvae at 2 dpf (Figure 3E; Tukey post-hoc pairwise comparison at 2 dpf: CTRL-1 µM VPA 24 h t_(236)_ = 2.464, *p* = 0.0235; CTRL-1 µM VPA 48 h t_(236)_ = 3.112, *p* = 0.0059). In TU strain larvae, we also observed a significant effect of treatment on the expression levels of *5htr3b* at 2 dpf (Figure 3F; Tukey post-hoc pairwise comparison at 2 dpf: CTRL-1 µM VPA 48 h t_(236)_ = 5.507, *p* < 0.0001; 1 µM VPA 24 h–1 µM VPA 48 h t_(236)_ = 3.642, *p* = 0.0010). For *5htr*4 and *sert*, we did not observe any effect of treatment in AB or TU strain larvae (Figure 3G,H,I,J, respectively).

**Figure 4:**
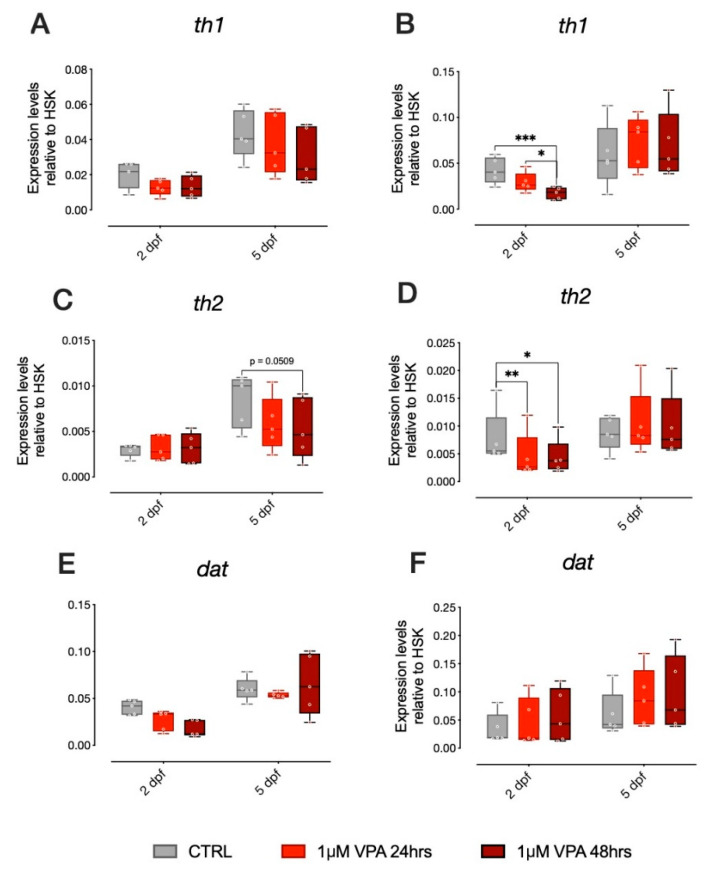
mRNA expression of key genes of dopaminergic system in 2 dpf and 5 dpf AB and TU zebrafish after 24 or 48 h of 1µM VPA. Changes in the expression of *th1* (**A**,**B**), *th2* (**C**,**D**) and *dat* (**E**,**F**) in AB (**A**,**C**,**E**) and TU (**B**,**D**,**F**) zebrafish embryos. Box plot (median, min to max) of mean dCt values for each group. **** *p* < 0.0001, *** *p* < 0.001, ** *p* < 0.01, * *p* < 0.05.

In addition to several reports on the effect of VPA on the serotonergic system, studies on zebrafish have shown that VPA treatment also affects key genes in other monoaminergic systems, in particular dopamine [19]. We thus analyzed genes involved in dopamine synthesis and metabolism in zebrafish larvae from the two different strains treated with our administration regimens (1 µM VPA for 24 or 48 h). Expression of *th1*, *th2* and *dat* were analyzed and statistical evaluation of the changes in gene expression did not reveal any significant effect of treatment in *th1* and *dat* in the larvae of the AB strain (Figure 4A,E, respectively), although an effect of VPA treatment on *th2* expression was observed (Figure 4C; Tukey post-hoc pairwise comparison at 5 dpf: *th1*, CTRL-1 µM VPA 48 h t_(236)_ = 2.351, *p* = 0.0509). In the TU strain larvae, however, we found a significant effect of treatment on the expression of both *th1* and *th2*, but not on *dat* (Figure 4B,D,F, respectively; Tukey post-hoc pairwise comparison at 2 dpf: *th1*, CTRL-1 µM VPA 24 h t_(236)_ = 4.122, *p* = 0.0002; CTRL-1 µM VPA 48 h t_(236)_ = 2.477, *p* = 0.04; *th2*, CTRL-1 µM VPA 24 h t_(236)_ = 3.010, *p* = 0.0081; CTRL-1 µM VPA 48 h t_(236)_ = 2.851, *p* = 0.0131).

## 4. Discussion

The administration of VPA in zebrafish represents a powerful model to study neurobiological and molecular changes relevant for ASDs. However, the use of heterogeneous experimental paradigms (dose and time of administration) limit comparison between studies, hindering the use of zebrafish models to investigate the neurobiological mechanisms underlying ASDs.

In order to identify the doses and administration regimens that had the lowest neurotoxic effects, we extended previous studies and tested micromolar doses of VPA, analyzing the animal survival rates and the expression of neurodevelopmental genes in two different wild type inbred strains: the AB and TU. We exposed AB and TU zebrafish embryos at 5 hpf to 1, 2.5 and 5 µM of VPA for 24 h and followed their survival rates for 5 days. We found that VPA already had a significant toxic effect at 1 µM concentration on both strains. We also characterized the effect of exposure to 1 µM VPA for 24 or 48 h on the expression of genes involved in neurodevelopment and known to impact neurotransmitter phenotypes.

The survival data in our study was in line with previous reports [22], since 1 µM VPA produced an increase in mortality in both AB and TU zebrafish strains. The discrepancy among our study and previous studies that did not report neurotoxic effect of VPA at higher concentrations, may be explained by the genetic differences of the inbred strains used in the studies. Zimmermann et al. [15] did not report the strain of the animals used in their study, while Baronio et al. [19] used the Turku strain. This study reported a high mortality rate (above 50%) in larvae treated with 50 µM, a high level of deformities and arrested development using 35 µM VPA, while no significant effects on mortality were reported by 25 µM VPA treatment [19]. Our data is also supported by Li et al. [22], who used a mixed AB-TU strain. Indeed, Li et al. [22] showed that, already at concentration ranges between 1 and 10 µM, VPA induced malformations in the endocrine and exocrine pancreas and the liver, also affecting craniofacial cartilage formation and vascular development.

We analyzed the expression of several neurodevelopmental genes at 2 and 5 dpf and observed an overall effect of age, independent of treatment. As expected, neurodevelopmental programs shape neural circuits in the first 5 days of development, affecting the expression levels of many genes. Globally, gene expression seemed to be mostly affected by VPA at 2 dpf in the TU strain larvae. Instead, for the AB strain, we observed gene expression changes both at 2 and at 5 dpf.

Most importantly, we observed striking differences induced by VPA in the expression of some of the genes between AB and TU strains. Given the equivalent effect of VPA at 1 µM concentration on survival rate in the two strains, we hypothesize that different genetic programs may activate distinct gene expression pathways in the two strains. Further studies, extending the set of genes analyzed through whole transcriptome sequencing, may be necessary to obtain a deeper understanding of the molecular programs that are affected by VPA in the AB compared to the TU strain.

Expression of the early neurogenic genes, *ascl1a* and *ascl1b*, was only affected by VPA in the AB strain. Previous reports analyzed the effect of HDAC inhibitors on neurogenic programs, showing the prominent role of HDAC in coordinating transcription and regulating cell fate in the developing nervous system through *ascl1a/b* [23]. HDAC inhibition mediated by VPA, directly inhibits *ascl1b*, causing the selective failure of serotonergic identity [18]. A similar effect was observed only in the AB strain larvae exposed to 1 µM VPA at 5 dpf, but not in the TU larvae, suggesting a difference in this specific neurodevelopmental program in the two strains. Despite the fact that VPA did not affect *ascl1a/b* expression in TU larvae, we still found significant changes in serotonergic markers in this strain. This data suggests that VPA may be sufficient to alter the serotonergic system development, independent of the genetic background, but indicates that different programs could be exploited to obtain similar effects on the serotonergic system in the two strains. Inbred strains may differ substantially due to inbreeding and genetic drift [24]. We tested two inbred, commercially available strains and showed that VPA exposure affected different molecular pathways in the two strains, suggesting different susceptibility to the drug and/or different mechanism of action.

Previous studies have analyzed the effect of VPA exposure on other monoaminergic system development, including the dopaminergic system [19], demonstrating a reduction of *th1* expression. We confirmed previous data with our dosage and administration regimens, analyzing the expression of *th1*, *th2* and *dat* in both strains. *th1* and *th2* expression was significantly affected by VPA only in the TU strain, although an effect of VPA treatment on *th2* expression was also observed also in the AB strain (Figure 4C). This data further highlights the potential differential effect of VPA on larvae of the two strains.

Recent studies have revealed alterations in the dopaminergic systems of mice embryonically exposed to VPA [25]. Epidemiological and animal model studies have also suggested that perinatal alterations in 5HT, either above or below typical levels, may cause social behavioral deficits resembling ASDs [26].

The lack of behavioral tests and detailed neuroanatomical analyses represents a limit to the interpretation of the molecular mechanisms underlying the neurotoxic action of VPA at micromolar doses used in this study. Previous reports have shown that zebrafish larvae do not exhibit social behavioral responses until 3 or 4 weeks of age [16,27], limiting the possibility to test social behavior at early stages. Future studies will determine whether low doses of VPA have long-lasting effects on social behavior and locomotor activity.

## 5. Conclusions

The zebrafish is emerging as a popular model in biomedical research to study the effect of neurotoxicants involved in nervous system disorders. To shed light on the toxic effect of VPA, we exposed zebrafish larvae of two different zebrafish inbred strains to 1 µM VPA for 24 and 48 h and tested embryo survival rates and changes in the expression of neurodevelopmental genes. Our results show that exposure to 1 µM VPA mildly affects embryo survival but is sufficient to induce changes in gene expression, with substantial differences between strains.

Further studies will be necessary to investigate the impact of VPA on neurodevelopmental mechanisms relevant to ASD pathogenesis. Several studies have indicated that VPA affects genes asymmetrically expressed in the brain of many vertebrate species (*5htr3a, 5htr3b* and *5htr4*) [21,28,29], including zebrafish, suggesting a potential role of this substance in the alteration of brain asymmetry associated with ASDs [30].

## Supplementary Materials

The following are available online at https://www.mdpi.com/2218-273X/10/10/1364/s1, Table S1: List of primers used for qPCR. Primers for *th1* and *th2* according to Baronio et al. [18], 18S, *htr3a*, *htr3b*, *htr4* according to Messina et al. [20].
