# Supplementary material for: Micromolar Valproic Acid Doses Preserve Survival and Induce Molecular Alterations in Neurodevelopmental Genes in Two Strains of Zebrafish Larvae
Source: Biomolecules. 2020 Sep 24;10(10):1364. doi: 10.3390/biom10101364 (PMC7601180; doi:10.3390/biom10101364)
Supplement: Supplementary file 1 [file biomolecules-10-01364-s001.pdf]

Table 1

Gene name	Gene ID	primer name	Primer sequence	amplicon length
tph2	407712	tph2 For	TATGACCAACAGCACCTTGC	138 pb
		tph2 Rev	GCTTTCACCAGAAAGCCAAC	
th1	30384	th1 For	GACGGAAGATGATCGGAGACA	94 pb
		th1 Rev	CCGCCATGTTCCGATTTCT	
th2	414844	th2 For	CTCCAGAAGAGAATGCCACAT	113 pb
		th2 Rev	ACGTTCACTCTCCAGCTGAGTG	
ascl1b	17172	ascl1b For	TCAGGTGCTTCAATGGCTAC	115 pb
		ascl1b Rev	TTGCTCAGTCAACGAACTGG	
ascl1a	30478	ascl1a For	CTGGGCAGTCCAAAGAAAAC	109 pb
		ascl1a Rev	TGAACTGCTGCTGGTTTACG	
htr3a	571641	htr3a For	TCGCTCAGCACAATGAGAAG	86pb
		htr3a Rev	TTCACTGAGCAATCCACCAC	
htr3b	571632	htr3b For	AGTGAGCGAAGTGGATTTGG	83pb
		htr3b For	GCTCCCATTCTCCATCATTC	
htr4	101882850	htr4 For	TAGTTTGCGCTGACAGCAAC	83pb
		htr4 Rev	AAGCTTTACCGGGTTTACGG	
SERT	664719	SERT For	ACATTTGCAGGTCTGGAAGG	90pb
		SERT For	GCCAAGCACAAACCATTCTC	
DAT	80787	DAT For	TCCTGCACAAACATCGAGAG	112pb
		DAT Rev	TGGTCCAACAGCGTAAACAC	
18S	100037361	18S For	TCGCTAGTTGGCATCGTTTATG	85 pb
		18 S Rev	CGGAGGTTCGAAGACGATCA	
